# Hereditary Transthyretin-Related Amyloidosis: Genetic Heterogeneity and Early Personalized Gene Therapy

**DOI:** 10.3390/biomedicines10102394

**Published:** 2022-09-25

**Authors:** Ketty Dugo, Francesca Bruno, Valentina Sturiale, Desiree Brancato, Salvatore Saccone, Concetta Federico

**Affiliations:** Department Biological, Geological and Environmental Sciences, University of Catania, Via Androne 81, 95124 Catania, Italy

**Keywords:** *TTR* gene, familial amyloidotic polyneuropathy, somatic mosaicism, gene therapy, amyloidosis, missense mutation, dominant genetic disease, Sicilian *TTR* mutation

## Abstract

Point mutations of the *transthyretin* (*TTR*) gene are related with hereditary amyloidosis (hATTR). The number of people affected by this rare disease is only partially estimated. The real impact of somatic mosaicism and other genetic factors on expressivity, complexity, progression, and transmission of the disease should be better investigated. The relevance of this rare disease is increasing and many efforts have been made to improve the time to diagnosis and to estimate the real number of cases in endemic and non-endemic areas. In this context, somatic mosaicism should be better investigated to explain the complexity of the heterogeneity of the hATTR clinical features, to better estimate the number of new cases, and to focus on early and personalized gene therapy. Gene therapy can potentially improve the living conditions of affected individuals and is one of the central goals in research on amyloidosis related to the *TTR* gene, with the advantage of overcoming liver transplantation as the sole treatment for hATTR disease.

## 1. Introduction

Hereditary amyloidosis TTR-related (hATTR) is a rare disease associated with several point mutations in the *transthyretin* (*TTR*) gene, determining abnormal aggregations of the transthyretin protein in different organs; hATTR is often misdiagnosed, as it is characterized by heterogeneous clinical features, including polyneuropathy, cardiomyopathy, vitreous opacity, and nephropathy, common to other diseases [1,2,3,4,5,6,7,8,9]. The history of amyloidosis transthyretin-related (ATTR) was traced back to 1929 to assess the impact of this rare disease in Europe. Reliable data exists, providing a clear picture of the epidemiology in several countries in the European Community. Over time, new diagnostic and therapeutic approaches were adopted, reducing misdiagnosis and contributing to the quality and life expectancy of the affected patients [10,11,12,13].

The most common point mutations of the *TTR* gene and the closely related clinical features of hATTR have been widely studied. Recently, an abnormal single nucleotide change in the fourth exon was described in a large family in Sicily (Italy) [14]. This was identified by sequence analysis using the Sanger method. Typically, genomic DNA for this type of analysis originates from whole blood, and the genomic DNA can be analyzed by various molecular strategies, not only through direct sequencing, but also using RFLP, southern blot, or other methods, depending on the type and the site of the mutation under analysis. Other tissues, such as buccal cells or hair bulb cells, could be used to obtain genomic DNA, avoiding the more invasive method of blood withdrawal and improving the availability of the patients [14,15,16,17,18,19,20]. The analysis of DNA from tissue other than the blood cells should be performed with caution, as it is possible that somatic mosaicism of the analyzed genomic region can be present in subjects with hATTR [14].

In this review, we describe the distribution of the *TTR* gene variants related to hATTR in Europe, and more specifically, in Sicily (Italy) and focus our attention on the role of somatic mosaicism in the *TTR* gene and its impact on the clinical variability of hATTR diseases, on early diagnosis, on future personalized therapies, and on a better estimating of cases in Europe and the world [14,20].

## 2. *TTR* Gene Variant and Hereditary Amyloidosis in Europe

**Figure 1:**
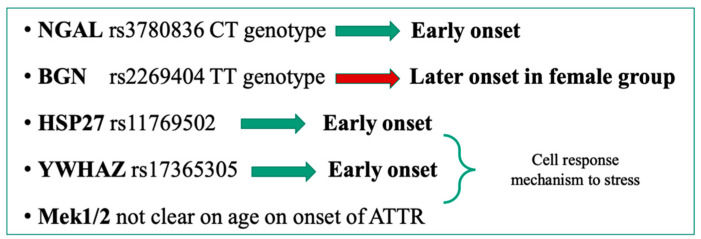
Genes and their SNPs involved in the genetic modulation of hATTR disease. The main gene, *TTR*, responsible for disease severity could be modulated by the presence/absence of specific alleles in the indicated genes. Data from [30].

Many cases of pathological transthyretin variants distributed in Europe were diagnosed and well-described in individuals of Italian origin, like the Val30Met, Gly47Ala, Thr49Ala, Phe64Leu, Tyr78Phe, and Glu89Gln variants [3,10,21,22,23,24]. Typically, transthyretin variants occur in heterozygosity and are usually transmitted as an autosomal dominant trait. However, homozygosity was found for Phe64Leu by means of gel electrophoresis, which showed an uncut fragment with a homozygous genotype [11,23]. Homozygosity and heterozygosity for other TTR point mutations were found in several studies [25,26]. Still, the impact is unclear for both clinical phenotypes and on the wide range of disease onset age in patients affected by the same or different TTR variants [27,28,29]. However, other genes could modulate the early or late age of onset (Figure 1), thus increasing the level of variability in this disease [30,31].

Compound heterozygosity in the *TTR* gene, such as Thr59Lys-Arg104His and Val30Met-Arg104His, was described in patients with much higher plasma concentrations of TTR and RBP (retinol binding protein) than in other patients only heterozygous for Arg104His [32,33]. This suggests nonprotective effects of the above compound heterozygosity in the progress of the disease. In another study on 129 hATTR patients, it was described that the most common mutations in *TTR* gene were:

- Val30Met   (c.148G>A)
- Phe64Leu   (c.250 T>C)
- Glu89Gln   (c.325G>C)
- Thr49Ala   (c.205A>G)

While other less frequent mutations were:

- Ala120Ser   (c.418G>T)
- Ile68Leu   (c.262A>T)
- Val122Ile   (c.424G>A)
- Ala109Ser   (c.385G>A)
- Thr59Lys   (c.236C>A)

In the same study, the phenotypic heterogeneity among the patients affected by TTR amyloidosis was confirmed and hypothesized the impact of the non-coding variants in the regulation of the *TTR* gene expression in source and target tissues as one of the mechanisms involved in this evident phenotypic heterogeneity [34].

## 3. Variability and Complexity of hATTR in Sicily (Italy)

The amyloidosis TTR-related symptoms are also determined by unclear mechanisms apart from mutations in the *TTR* gene and in some other known genes (see Figure 1). Since 1:100,000 Italians are affected by this rare disease, further research is necessary to obtain data that are not underpowered [34,35]. In Italy, it is likely that cases of hATTR remain underestimated, as well as some TTR pathological variants being poorly documented in terms of geographical distribution and the clinical characterization of severely affected individuals. Genetic anticipation of serious symptoms, including an early age of onset and the rapid death of the subjects, was described for hereditary amyloidosis related to the Gly47Glu variant of the *TTR* gene. The genetic analysis showed a transition G>A on exon 2 of the *TTR* gene, where this point mutation caused the change of glycine with glutamate in position 47. This change seems to alter the normal monomer folding and to promote an easier TTR amyloidotic deposition in the target organs, possibly related with the severity of clinical features. However, other unclear genetic and environmental factors seem to have a role in the onset of severe symptoms of the disease [7,8].

**Figure 2:**
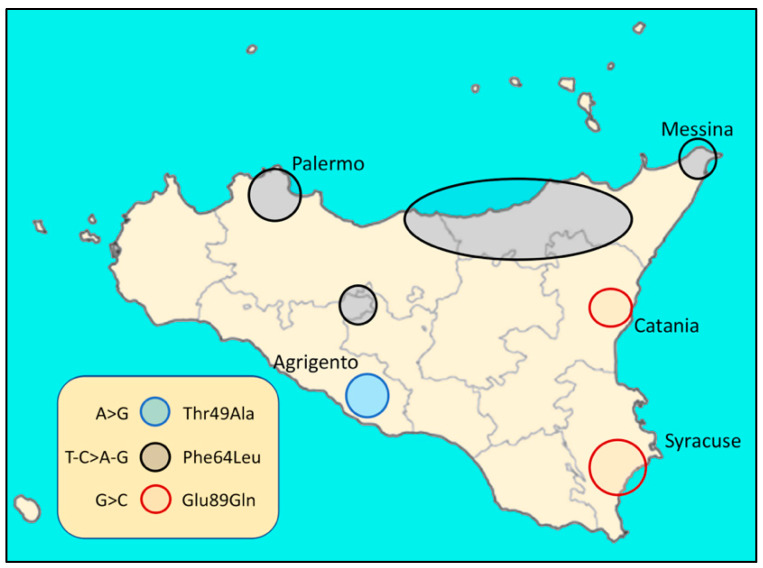
Distribution in Sicily (Italy) of the three endemic TTR variants related to FAP.

**Figure 3:**
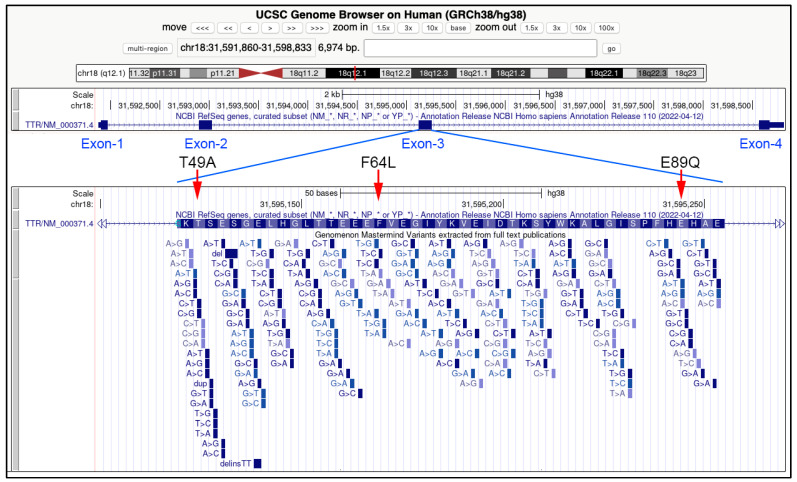
Genomic features of the three endemic TTR variants related to FAP and described in Sicily (Italy). In the upper part of the image, the *TTR* gene, spanning about 7000 bp in the 18q21.1 chromosomal band, and the position of the four exons. In the bottom panel, an enlargement of the exon three, with the position of the three Sicilian genetic variants: T49A (Thr49Ala), F64L (Phe64), and E89Q (Glu89Gln). All the variants in the third exon described in the scientific literature are indicated below the aminoacidic sequence corresponding to the coding sequence of this exon. Data and images are from the UCSC Genome Browser (http://genome.ucsc.edu, accessed on 26 August 2022).

In Italy, hATTR cases have been described in several regions [36]. For example, in Sicily (Italy), three endemic TTR variants were associated with FAP (familial amyloidotic polyneuropathy) (Figure 2 and Figure 3):

Glu89Gln (c.325G>C)

Thr49Ala (c.205A>G)

Phe64Leu (c.250 T>C)

To date, all analyzed Sicilian patients were heterozygous for the above mutations and no homozygous TTR variant was found [10,37,38]. However, the presence of rare homozygous individuals for amyloidotic TTR variant cannot be ruled out. Further investigations could clarify this. The preliminary estimate from the first Sicilian hATTR epidemiological study was 8.8/1,000,000 cases, though this value is likely to increase [23,37].

In 1992, a point mutation G>C in exon 3 of the *TTR* gene was found linked to an aminoacidic change from glutamate (GAG) to glutamine (CAG) in the TTR protein [3]. Previously, a new dominant TTR mutation was found to be related to sensory and peripheral neuropathy, vitreous opacities, and cardiomyopathy, with an early age of onset of disease [39]. In another study, screening tests on serum samples from 74 patients with different TTR mutations and DNA analysis by isoelectric focusing (IEF) showed Glu89Gln and other TTR variants causing hATTR [40]. It was shown that many individuals are heterozygous for Glu89Gln. A founder effect could explain the occurrence of Glu89Gln TTR variants in many Bulgarian individuals, for whom the age of onset is comparable to Sicilian hATTR patients. However, TTR-related cardiomyopathy in Bulgarian patients is worse than that of the Sicilian cohort, and it seems that the clinical phenotype is strongly influenced by respective geographic area. Other TTR pathological variants related to hATTR were described in 2008 in Bulgaria [37,41,42,43,44]. However, further studies could elucidate on the heterogeneity of clinical features and molecular events that occur in modulating the severity and progression of the disease.

## 4. First Case Report of Somatic Mosaicism in hATTR Patients and Gene Conversion

Recently, a somatic mosaicism was discovered, disclosing a reversion to the normal TTR Glu89Gln (NM_000371.3:c.325G>C) variant. Thus, it would be appropriate to run genomic DNA testing from other tissues in case of negative results from whole blood for all patients with a family health history of hATTR, and it would be interesting to know on what molecular basis somatic mosaicism occurs and how this could affect the clinical features and severity of hATTR disease. Other TTR variants could be identified in genomic DNA from blood, but also from buccal cells or hair of the same patient [14]. The reverting of a pathogenic mutation to normal could represent a new concept of natural gene therapy previously highlighted for other inherited diseases [45]. In humans, it was first demonstrated that a mitotic gene conversion occurred as the genetic mechanism causing revertant mosaicism in a case of a milder clinical phenotype of epidermolysis bullosa [45]. Several mechanisms were proposed to explain a reversion to normal of a DNA sequence, including intragenic recombination, mitotic gene conversion, or second site compensating mutations [46]. A subsequent study supported that double-strand breaks (DSBs) were the initial events of gene conversion and, moreover, how many molecular substrates are necessary for recombinant-repair processes [47]. Understanding gene conversion mechanisms in eukaryotic genome evolution and in inherited diseases can clarify some unclear aspects on the biological answer of an organism to the occurrence of a genetic disease [48,49,50,51]. Gene conversion was demonstrated for the TTR Val30Met variant in vitro and in vivo but is still very far from clinical application [52]. However, it was proposed as a possible gene therapy among the new molecular approaches for treating hATTR [53]. Several known molecular mechanisms can lead to a spontaneous reversion of a gene mutation, causing somatic mosaicism in rare human disorders with more tolerable clinical phenotypes. However, it was highlighted that these phenomena should be sustained by more detailed genetic and molecular analysis, and genomic DNA should be obtained from blood and other tissues, such as buccal mucosa, not forgetting the impact on clinical and ethical aspects [54]. In this regard, several individuals affected by hATTR should be screened to verify the presence of reversion to normality of an amyloidotic *TTR* gene variant, as observed for Glu89Gln. The clinical impact of this event was not demonstrated but it was observed that a higher rate of individuals from the IV generation did not inherit the Glu89Gln TTR mutation if a parent carried the reversion CAG > GAG in some analyzed tissue sample [14]. Furthermore, in the IV generation, individuals carrying the same form of somatic mosaicism were present in a single family branch.

**Figure 4:**
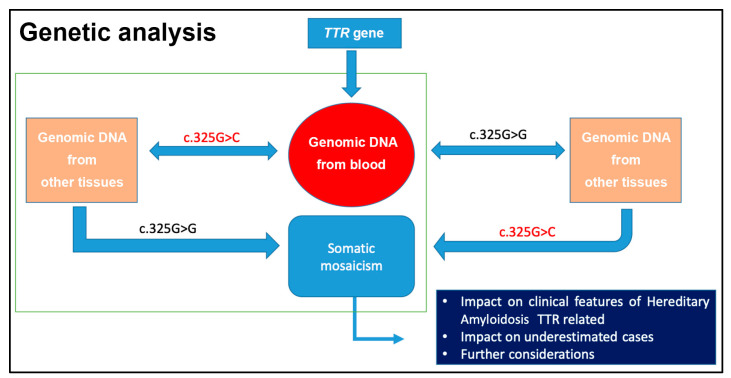
Relevance to analyze genomic DNA from several tissues, as in the case of G>C mutation in the *TTR* gene (NM_000371.3:c.325G>C) [14].

Somatic mosaicism could influence the transmission of an autosomal trait; however, such could be due to many other issues, including other gene mutations (Figure 4). In 2001, an interesting review showed that somatic mosaicism is very important to evaluate the genetic heterogeneity in several monogenic, neurodegenerative, and complex diseases. More in-depth genetic analyses, including more than one type of tissue, are needed. The human genome is not stable and homogeneous, and this concept is fundamental in the genetic counseling context [14,55,56,57,58].

It has been reported that the expression of the *TTR* gene varies over time and that there is a difference in the ratios between wild-type and mutated transcripts that often correlate with the age of onset and with the penetrance of the disease. Liver biopsy remains the golden standard for transcriptional analysis of the *TTR* gene; however, the use of plasma and urine are less invasive methods for relative quantifications between mutant and wild-type transcripts. Wild-type peaks in each sample were used as normalizers and considered to be equal to 1 unit, and the mutated alleles ranged from 0.11 to 1.14 units. In this way, a monoallelic expression on both plasma and urine was surprisingly highlighted. From the processed samples, 87% showed monoallelic expression and 13% biallelic expression in plasma, while in urine 58% showed monoallelic expression and 42% biallelic expression. Furthermore, it was observed that, at an early age, there is a predominant expression of the wild-type form, while the expression of the mutated allele increases with advancing age; when the onset of the disease is around the age of 50, it is possible that there is a biallelic expression, until there is some sort of natural selection or suppression, which favors the expression of the mutated transcripts [59].

The case of a father who passed on p.Glu89Gln to both his twin sons is interesting; one of the two sons presented symptoms before his father and 11 years before his twin. Clearly, additional factors seem to play a fundamental role on the age of onset and the severity of the disease. Investigating the presence of somatic mosaicisms and how these can affect the expression of the *TTR* gene in different tissues could be useful to understand their role on the expressiveness and on the penetrance of the pathology. Patients with the missense mutation G>C, responsible for the amino acid substitution p.Glu89Gln, in the DNA extracted from blood but not in DNA extracted from buccal or hair bulb cells, have manifested the disease. These patients have not had transcriptional analysis of the *TTR* gene [14,59,60]. However, the involvement of other unidentified genes in the modulation of *TTR* gene expression is very likely.

## 5. Gene Therapy and ATTR

Conversion from the pathogenetic mutations of the *TTR* gene to the wild-type variant was observed in vivo in DNA of buccal and hair bulb cells [14]. This gene conversion toward the wild-type version of the gene was proposed for future genetic therapy in spite of the limitations, such as the efficiency of conversion in target tissues, the drug delivery, the safety, and the costs. Tafamidis is a stabilizer of the TTR tetramer, but TTR could be stabilized by a new method that involves gene therapy by introducing a non-amyloidogenic variant of the TTR monomer (T119M) previously found in Portuguese individuals with milder symptoms. Thus, a protective TTR variant could stabilize the TTR tetramer and possibly block amyloidotic progression. This approach could be used to prevent disease or to control symptom onset [61,62,63,64].

**Figure 5:**
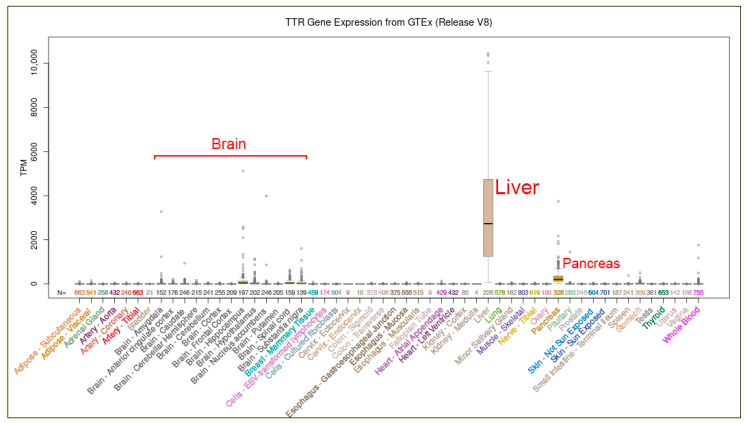
Expression level of the *TTR* gene in 54 healthy human tissues from GTEx RNA-seq of 17,382 samples from 948 donors (V8, Aug 2019). TPM: transcripts per million. Data and images from the UCSC Genome Browser (http://genome.ucsc.edu, accessed on 26 August 2022).

Liver transplantation was the first therapy for hATTR, but as it is not always effective, several drugs have been developed in an attempt to delay symptoms and improve patient life span. It seems that the choice of therapeutic options is closely associated to a given diagnosis of hATTR and depends on the overall condition of the patient, while also evaluating the cases observed in non-endemic areas [53,65,66,67,68]. Silencing the *TTR* gene by RNA interference seems hopeful, but several factors and other genes can improve the age of onset and the progression of the disease. Reverting amyloidogenic TTR mutations to the wild-type profile could be a new approach for treatment of the diseases but conversion to normality is not possible in all tissues expressing the *TTR* gene, with the liver being the major organ of TTR gene expression (Figure 5). In this regard, the question remains open whether the somatic mosaicism can ameliorate the clinical features and life span of patients with hATTR [14,52,69,70,71,72,73].

From a study also conducted in Italy, it was found that the patients affected by hATTR who will benefit from the treatment with Patisiran will be more and more numerous. New scientific evidence indicates that both neurological and cardiological conditions of transplanted patients appear to be worsening. Liver transplantation aims to suppress the main source of the mutated protein; however, even in minimal quantities, TTR is produced by other organs such as the brain, the retina, and the pancreas (Figure 5). Transplantation demonstrated greater efficacy and more satisfactory results in subjects with early onset of the Portuguese variant (Val30Met). However, in Italy and in other European countries, this mutation is associated with a later onset and a different phenotype, and it has been observed that in these affected populations, liver transplantation is less effective, also because of the continuous deposition of the wild-type transthyretin on previously formed amyloid deposits, leading to further progression of the disease. The use of Patisiran interfering RNA molecules was also evaluated on patients with hATTR who presented disease progression despite having performed an orthotopic liver transplant. Patisiran therapy induced a reduction in TTR serum levels of more than 80%, and an improvement in polyneuropathy and overall quality of life in this cohort of patients, consistent with the results previously conducted in non-transplanted patients on which the efficacy and safety of these molecules were studied and observed [74,75].

The *TTR* gene represents an ideal target for the application of gene editing mediated by Crispr-Cas9 in the treatment of hATTR. In fact, the knockout of this gene would produce, in addition to an improvement of the disease that is due to the lack of further deposition of the protein in the target organs, physiological effects limited to the transport of thyroxine and vitamin A [76]. It has been reported that the systemic administration of the NTLA-2001 molecule reduced the serum concentration of TTR in six patients with hATTR. This dose-dependent reduction varied from 80% to 96% [77].

## 6. Conclusions

Diagnosing hATTR is often very difficult, especially in the early stages when the symptoms are absent or unclear or overlap with other clinical conditions. Diagnosis is even more problematic when the family history is unknown, and even then it is difficult to assess the best time to perform the genetic test, particularly in very young individuals. In the case of positivity to a specific pathogenetic variant, the pharmacological treatment in the asymptomatic subject must be carefully evaluated in terms of risk/benefit. The pathology does not affect the fitness of the affected individuals and it is for this reason that in genetic counseling it is appropriate not to underestimate the reproductive risk. Although the molecular mechanisms are partially known, it would be useful to investigate possible factors that may also influence the response to pharmacological treatments, and to evaluate the combination of multiple treatments in patients with an advanced stage of the disease or in patients who do not fully respond to treatment with interfering molecules [78,79,80].

The best treatment for patients affected with hATTR is closely related to an early diagnosis. Many cases of hATTR are underestimated, especially in non-endemic areas where family history of the disease is not available, and because clinical features often overlap with those from other diseases. Indeed, it is very difficult to predict the age of disease onset. More than 100 points of mutations of the *TTR* gene are involved in hATTR, while other factors may be involved in the complex mechanism of progression of the disease, in the heterogeneity and the variability of the symptoms, the transmission, the life span, and in the response to new therapies [10,81]. Somatic mosaicism in hATTR could have a role in ameliorating the patients general health, as previously described in other diseases.

The conversion from the amyloidogenic variant to the wild type in tissue expressing the TTR gene could influence the age of onset of the disease, as well as the interactions with other factors involved in its progression [48,49,50,51,52,53]. As an example, somatic mosaicism should be investigated also in negative subjects to ameliorate estimated endemic cases, especially in non-endemic areas. Somatic mosaicism in hATTR patients was observed in carriers of the Glu89Gln TTR variant. The effect of conversion to normal of a pathological point mutation of the *TTR* gene, disclosed by a somatic mosaicism in individuals affected by hATTR, is unclear. This phenomenon can also be observed in other families, and in this regard it would be interesting to evaluate a possible role in modulating symptoms and disease progression [14,54]. HATTR is a rare disease where many other factors should be investigated; mutations of the *TTR* gene are the major actors on the severity of the disease but secondary interactions could be the key for understanding other unclear pathways leading to severe disease [30,63]. Averting the drop-out allele phenomenon could improve the efficiency of diagnosis in both dominant and recessive pathologies. Checking the PCR products qualitatively and quantitatively is an important step to avoid false positives or false negatives [82].

Gene therapy and immunotherapy could potentially be the major treatments for hATTR. However, evaluating a possible somatic mosaicism could ameliorate the rate of the asymptomatic people affected in several countries and its role in hATTR should be further investigated toward a personalized clinical treatment [10,14,61,62,83].

## Data Availability

Not applicable.
